# Mass Occurrence of Anatoxin-a- and Dihydroanatoxin-a-Producing *Tychonema* sp. in Mesotrophic Reservoir Mandichosee (River Lech, Germany) as a Cause of Neurotoxicosis in Dogs

**DOI:** 10.3390/toxins12110726

**Published:** 2020-11-20

**Authors:** Franziska Bauer, Jutta Fastner, Bernadett Bartha-Dima, Wolfram Breuer, Almuth Falkenau, Christian Mayer, Uta Raeder

**Affiliations:** 1 Limnological Research Station Iffeldorf, Aquatic Systems Biology Unit, Technical University of Munich, Hofmark 1-3, 82393 Iffeldorf, Germany; uta.raeder@tum.de; 2 German Environment Agency, Schichauweg 58, 12307 Berlin, Germany; jutta.fastner@uba.de; 3 Bavarian Health and Food Safety Authority, Veterinärstraße 2, 85764 Oberschleißheim, Germany; Bernadett.Bartha-Dima@lgl.bayern.de (B.B.-D.); Wolfram.Breuer@lgl.bayern.de (W.B.); 4 Center for Clinical Veterinary Medicine, Institute for Veterinary Pathology, Ludwig-Maximilians-University, Veterinärstraße 13, 80539 Munich, Germany; falkenau@patho.vetmed.uni-muenchen.de (A.F.); mayer@patho.vetmed.uni-muenchen.de (C.M.)

**Keywords:** cyanobacteria, anatoxin-a, dihydroanatoxin-a, *Tychonema*, neurotoxicosis, cyanotoxins, macrophytes, benthic, tychoplanktic, reservoir

## Abstract

In August 2019, three dogs died after bathing in or drinking from Mandichosee, a mesotrophic reservoir of the River Lech (Germany). The dogs showed symptoms of neurotoxic poisoning and intoxication with cyanotoxins was considered. Surface blooms were not visible at the time of the incidents. Benthic *Tychonema* sp., a potential anatoxin-a (ATX)-producing cyanobacterium, was detected in mats growing on the banks, as biofilm on macrophytes and later as aggregations floating on the lake surface. The dogs’ pathological examinations showed lung and liver lesions. ATX and dihydroanatoxin-a (dhATX) were detected by LC-MS/MS in the stomachs of two dogs and reached concentrations of 563 and 1207 µg/L, respectively. Anatoxins (sum of ATX and dhATX, ATXs) concentrations in field samples from Mandichosee ranged from 0.1 µg/L in the open water to 68,000 µg/L in samples containing a large amount of mat material. Other (neuro)toxic substances were not found. A molecular approach was used to detect toxin genes by PCR and to reveal the cyanobacterial community composition by sequencing. Upstream of Mandichosee, random samples were taken from other Lech reservoirs, uncovering *Tychonema* and ATXs at several sampling sites. Similar recent findings emphasize the importance of focusing on the investigation of benthic toxic cyanobacteria and applying appropriate monitoring strategies in the future.

## 1. Introduction

Cyanobacteria are common components in aquatic systems. Phototrophic cyanobacteria are ubiquitously distributed, can form mass populations under eutrophic conditions and certain strains of many taxa can produce different toxins [1,2,3,4,5]. Therefore, cyanobacteria can cause major problems in drinking water supplies, fish farming and bathing waters. Cyanotoxins are among the strongest biogenic toxins found in nature [6,7]. The toxicity of cyanotoxins can be ranked between the tetrodotoxin of marine poisonous animals and strychnine from *Strychnos nux-vomica*, known as arrow poison [6]. According to their mode of action, cyanotoxins can be divided into hepatotoxins, neurotoxins and cytotoxins, leading to liver or respiratory failure after ingestion of hazardous amounts of toxins [6]. Beyond this, effects reported after contact with cyanobacteria include, e.g., odor impairments, skin reactions and gastrointestinal problems. These are, however, rather attributed to other metabolites from cyanobacteria or associated bacteria than to cyanotoxins.

In the past, it was assumed that cyanobacterial blooms occur mainly in eutrophic waters [8]. Numerous findings demonstrate that planktic cyanobacteria dominate in lakes with higher trophic levels and that blooms are more frequent there [9,10,11]. However, it is known that cyanobacteria colonizing the bottom of rivers and lakes can also be found in waters with lower trophic levels and that they can produce the same toxins as planktic cyanobacteria [12]. Nevertheless, the knowledge about benthic cyanobacteria in lakes and rivers is still very limited. To date, studies on toxic benthic cyanobacteria are often descriptive and based on case studies. These were often performed in the context of animal poisonings, which have been increasingly reported in recent years [12]. Until now, the European Bathing Water Directive (Directive 76/160/EEC concerning the quality of bathing water) regulates only the surveillance of planktic cyanobacteria and their threat to humans. 

For example, repeated incidents of dog poisoning occurred from toxic cyanobacteria in mesotrophic Lake Tegel (Berlin, Germany) not detected by standard surveillance protocols for bathing water. No planktic blooms were present at the time of the incidents. However, there was a mass occurrence of detached water moss (*Fontinalis antipyretica*), which was densely populated by the anatoxin-a (ATX)-producing cyanobacterium *Tychonema* [13]. This incident showed for the first time a problematic occurrence of tychoplanktic toxic cyanobacteria in a German lake. Therefore, Fastner et al. [13] requested a revision of the bathing water surveillance strategies at that time. 

Since the first incident in May 2017, ATX-producing cyanobacteria associated with macrophytes have occurred regularly in Lake Tegel and, most probably, other dogs died in 2019 due to ATX (Fastner et al., unpublished data). The question arises as to whether this problem is limited to Lake Tegel or whether toxic benthic/tychoplanktic cyanobacteria are to date simply undiscovered and underestimated in German inland waters. In general, however, the incidents at Lake Tegel have increased attention on such cases. Apart from *Tychonema*, other cyanobacterial genera known for their capability of producing ATX are *Anabaena*, *Aphanizomenon*, *Cylindrospermum*, *Microcoleus*, *Oscillatoria*, *Phormidium* and *Raphidiopsis* [14,15,16,17,18,19,20,21,22].

In August 2019, three dogs died after bathing in or drinking from Mandichosee, a reservoir of the River Lech (Bavaria, South Germany). Intoxication was suspected and, in particular, poisoned baits were presumed, but poisoning by toxic cyanobacteria has also been taken into consideration. Aware of the incidents at Lake Tegel, the aim of the present study was to reveal the causes of the dog deaths by means of a sampling campaign initiated immediately after the incidents at Mandichosee. 

Surface algal blooms were not visible in Mandichosee at the time of the incidents. Instead, there were red-brown mats on the shore areas as well as macrophytes in the shallow water densely covered with red-brown biofilms. Later, red-brown aggregations were also floating at the lake surface in the shore areas. Microscopy revealed mass abundances of benthic *Tychonema* sp. Toxin analyses were conducted from the dogs’ stomachs, and different environmental samples. Furthermore, a molecular approach was used to detect toxin genes by PCR and to reveal the cyanobacterial community composition by Illumina MiSeq sequencing. In order to better estimate the extent of the distribution of benthic *Tychonema* sp., the Lech reservoirs were also sampled upstream. 

## 2. Results

### 2.1. Macroscopic and Microscopic Appearance of Tychonema

**Figure 1:**
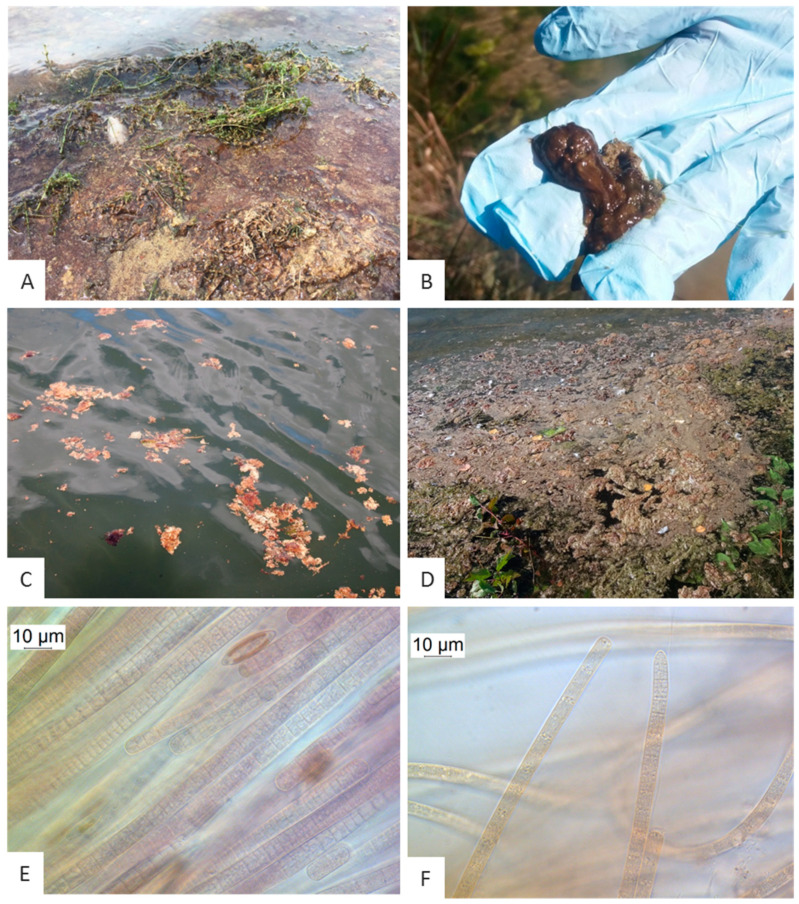
Appearance of benthic *Tychonema* sp. in Mandichosee (**A**–**C**,**E**) and River Lech upstream (**D**,**F**). (A) Red-brown biofilm at the shorelines and on dead plant material in Mandichosee. (**B**) Red-brown clumps floating on the water surface in Lechaue. (**C**) *Tychonema* aggregates floating on the water surface in Mandichosee. (**D**) Mass occurrence of *Tychonema* aggregates in the reservoir Schwabstadl. (**E**) Microscopy of red-brown biofilm taken from Mandichosee (×1000). (**F**) Microscopy of red-brown biofilm taken from Lechaue.

**Figure 2:**
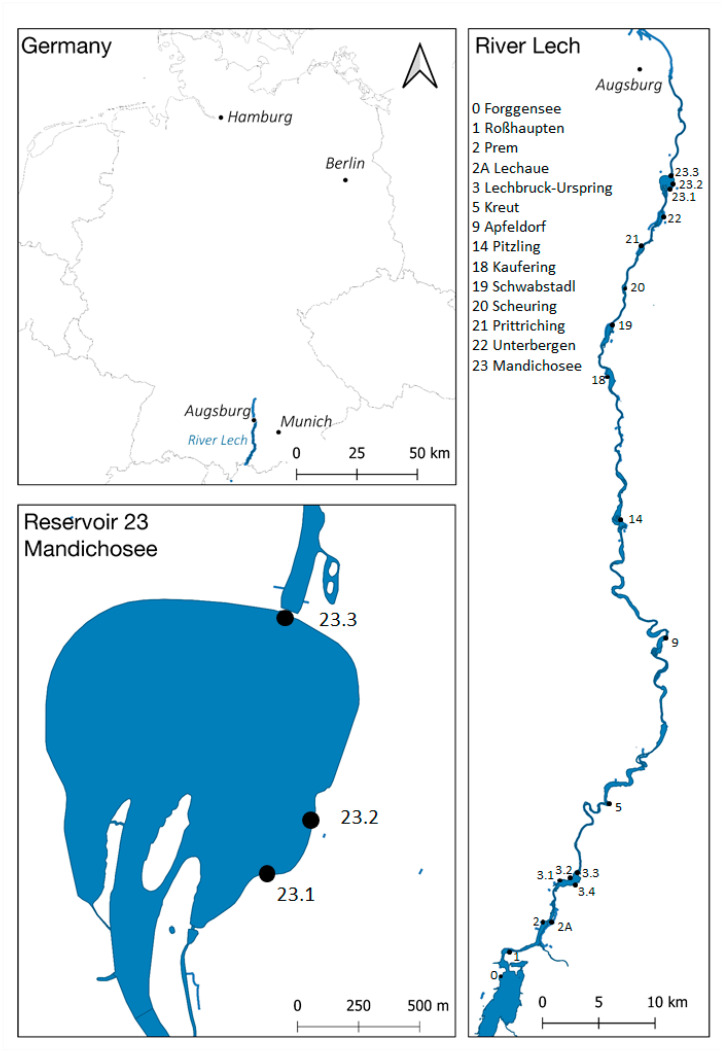
Map of the study area and the sampling sites (dots) in Mandichosee and River Lech. Site 23.1 is the bathing area where the dog casualties occurred. Built with QGIS (http://www.qgis.org), data/maps copyright: Geofabrik GmbH and OpenStreetMap Contributors (https://download.geofabrik.de/europe/germany/bayern.html).

Red-brown biofilms were detected on dead and living plant material of the macrophytes *Elodea*, *Potamogeton*, *Myriophyllum* and unidentified filamentous algae (Figure 1A) at the shorelines of the reservoir Mandichosee (Figure 2, sites 23.1–23.3). Furthermore, red-brown aggregations were found growing on the sediment (Figure 1A) along the shorelines (Figure 2, site 23.2). Near the bathing area and at the shores in the southern reservoir (Figure 2, site 23.1), red-brown slimy aggregates were floating at the surface from the second sampling date onwards (Figure 1C). 

Upstream of the River Lech, red-brown scums were also detected in different abundances in reservoirs Lechaue (2A), Lechbruck-Urspring (3.2 and 3.4), Kaufering (18), Schwabstadl (19), Scheuring (20) and Prittriching (21) (Figure 2). The shorelines of the reservoirs Schwabstadl (19) and Prittriching (21) were heavily covered with these scums (Figure 1D). In the reservoir Lechaue (2A), red-brown biofilm has also been found growing on dead wood.

The microscopic analyses of the biofilms of sampled plants, sediments and of the scums revealed filamentous cyanobacteria, which could be identified as *Tychonema* sp. based on their morphological traits (Figure 1E,F). Trichome widths varied between 5.5 and 14.0 µm. The trichomes of the *Tychonema* samples taken in August and early September 2019 in the northern waters between the reservoir Kaufering (18) and the reservoir Mandichosee (23) differed from those collected later in September 2019 from the southern reservoirs Lechaue (2A) and Lechbruck-Urspring (3): centripetal cross walls were clearly visible in northern samples (Figure 1E), whereas filaments were less structured in the southern samples and rather showed granulated cross walls (Figure 1F). An overview of the microscopic results is shown in Table S1 (Supplementary Material).

### 2.2. Animal Necroscopy and Histopathology

The symptoms of two dogs (D1 and D2) had been retching, salivation and dyspnoea. The third dog (D3) had shown acute seizures and oral discharge. Post-mortem examination of D1 and D2 revealed congested lungs and livers, accompanied by an edema of the wall of the gallbladder in the case of D1. In D3, the lung was diffusely solidified and the liver was moderately swollen. Histologically, the lungs of D1 and D2 showed slight congestion and edema, and in D3, severe pulmonary congestion, edema and mild diffuse interstitial pneumonia with type II pneumocyte hyperplasia, infiltration of interalveolar septa with macrophages, lymphocytes, plasma cells and single neutrophils and similar mild multifocal perivascular infiltration. The livers of D1 and D2 were mildly hyperemic. Sudan red stains were inconspicuous. The liver of D3 displayed some randomly scattered foci with minimal hepatocellular degeneration and resorptive infiltration. In D1, multifocal hemorrhage was seen in the brainstem. Furthermore, the stomach and intestine of D3 were affected by a mild multisegmental mucosal lymphoplasmacytic infiltration (mild gastroenteritis). Other organs showed no structural anomalies of inflammatory, degenerative, malformative or neoplastic origin.

### 2.3. Toxin Analyses

**Figure 3:**
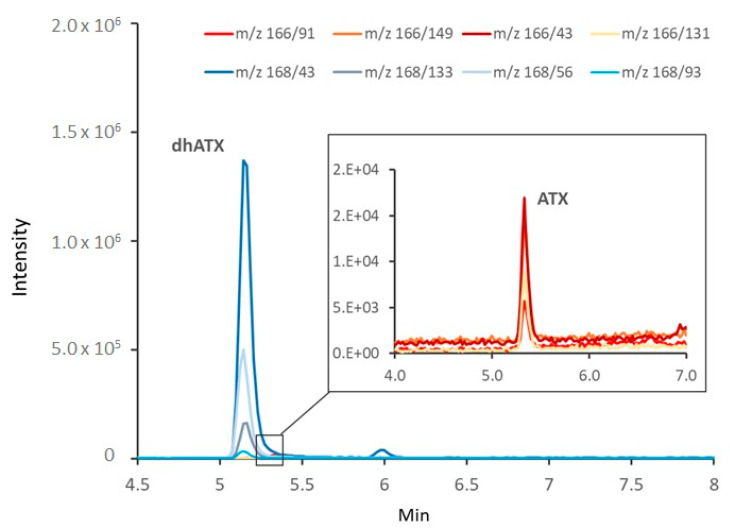
Reconstructed LC-MS/MS chromatogram from a sample from Mandichosee, 23 August 2019. Bluish lines represent transitions indicative for dihydroanatoxin-a (DhATX) and reddish ones, those for anatoxin-a (ATX).

DhATX and ATX were detected in nearly all samples containing *Tychonema* mats, macrophytes covered with *Tychonema* and sediments, as well as in the stomachs of the dogs (Figure 3, Table 1). In contrast, they could hardly be found in the open water, even near the mats (Table 1). Cylindrospermopsin (CYN), deoxycylindrospermopsin (Deoxy-CYN), homoanatoxin (HATX) and dihydrohomoanatoxin (DhHATX) were not detected in any sample.

The sum of ATX and dhATX in samples containing *Tychonema* (mats, macrophytes, sediments) ranged from 0.1 µg/L in the open water up to almost 68,000 µg/L in samples containing a very large amount of mat material (Table 1). Concentrations were overall higher in Mandichosee in August than in other reservoirs of the river Lech sampled in September. 

Contents of ATXs in *Tychonema* mat biomass ranged between 86 and 353 µg/g fresh weight (FW) (Table S2, Supplementary Material). Assuming a dry weight (DW) of 10% of fresh biomass, contents per DW were between 860 and 3537 µg/g. 

The concentration in the dogs’ stomachs was 563 and 1207 µg/L, respectively (Table 1).

### 2.4. Detection of Toxin Genes

None of the samples contained the target genes for microcystin or saxitoxin. The target gene for ATX *anaC* could be detected in the samples taken from Mandichosee next to the bathing area (site 23.1, on 21 August 2019), next to the parking area (site 23.2, on 19 and 21 August and 10 September) and next to the power plant (site 23.3, on 19, 21 August and on 10 September 2019). Furthermore, *anaC* genes were detected in scum samples taken on 12 September 2019 upstream of Mandichosee in the reservoirs Schwabstadl (19) and Prittriching (21) (Figure 2, sites 19 and 21). The results are summarized in Table S1 (Supplementary Material).

### 2.5. Illumina MiSeq Sequencing

The first sequencing run revealed a total of about 600,000 sequences, clustered into 407 bacterial operational taxonomic units (OTUs). Following the SILVA classification [23], the most frequently occurring OTU was assigned to the cyanobacterial genus *Tychonema*. Besides the *Tychonema* OTU, there were five other cyanobacterial OTUs: three OTUs classified as *Cyanobium*, and one OTU each attributed to the genera *Limnothrix* and *Pseudanabaena*. Furthermore, two cyanobacterial OTUs were not classified down to genus level but were assigned to the order Oxyphotobacteria and Caenarcaniphilales (Melainabacteria). The run of the second part of the samples revealed a comparable result. 

The samples from the reservoirs in Kaufering (18), Schwabstadl (19) and Scheuring (20) exclusively contained *Tychonema* as cyanobacterial OTUs. The *Tychonema* OTU was present in all samples taken from Mandichosee except the open water sample taken next to the power plant. Furthermore, the *Tychonema* OTU was present in the bank sample taken downstream of the power plant at dam 23, which forms the reservoir Mandichosee. In addition, *16S rRNA gene* sequences of *Tychonema* were found in several samples taken in the reservoirs of River Lech upstream. In all these samples, the presence of *Tychonema* was already suspected based on macroscopic examination and detected microscopically: Lechaue (2A), Lechbruck-Urspring (3.2, 3.4), Kaufering (18), Schwabstadl (19), Scheuring (20) and Prittriching (21). In the samples of reservoirs Prem (2), Kreut (5), Apfeldorf (9) and Unterbergen (22), *Tychonema* could only be identified by sequencing. Reservoirs Forggensee (0), Roßhaupten (1) and sampling site Pitzling (14) were tested negatively for *Tychonema* genes. An overview of *Tychonema*-positive and -negative sampling sites is given in Table S1 (Supplementary Material).

### 2.6. Hydro-Chemical and Hydro-Physical Characterization 

Hydro-chemical and hydro-physical parameters are summarized in Table S1 (Supplementary Material). The total phosphorus contents of 10 samples, which were taken from the site below the reservoir Pitzling (14) downstream to the reservoir Mandichosee (23) varied between 11.1 and 20.3 µg/L. These few phosphorus values give a first indication of the trophic status of water bodies, which can be classified between oligo-mesotrophic and mesotrophic according to Schneider et al. [24]. In all samples, the soluble reactive phosphorus was below the detection limit of 5 µg/L. The concentrations of inorganic nitrogen were only measured in the reservoir Mandichosee. The nitrate nitrogen content was 0.5 mg/L and the ammonia nitrogen content was 0.02 mg/L.

The hydro-physical parameters measured at different times along the shoreline of the reservoir Mandichosee were almost identical, except for oxygen saturation in regions with and without benthic *Tychonema* occurrence. The surface temperature varied between 14.3 and 19.8 °C, pH values between 8.4 and 8.7, and conductivity between 307 and 341 µS/cm. Oxygen saturation varied between 115 and 142%, with supersaturations indicating photosynthetic activity of *Tychonema* aggregations.

Additionally, the investigated sites in the other reservoirs of the River Lech where *Tychonema* was found were described hydro-physically. The temperature ranged from 11.8 °C to 19.3 °C. The pH values ranged between 8.1 and 9.4. The conductivity was between 324 and 494 µS/cm and thus tended to be slightly higher than in the reservoir Mandichosee. The oxygen saturation was between 96 and 177%. The high supersaturations were determined directly in the *Tychonema* aggregates. At these locations, an optically recognizable bubble formation already indicated the oxygen production by the primary producers.

## 3. Discussion

The filamentous cyanobacteria detected in mats from Mandichosee and the River Lech were identified as *Tychonema* due to their morphological traits [25]. This presumption was confirmed by molecular biological methods. The cyanobacteria filaments occurred as a benthic species and could also be found in the form of floating scums. Although the cell widths overlap with other *Tychonema* species, such as *Tychonema bourellyi* (4.0–6.0 µm) or *T. tenue* (5.5–8 µm), *Tychonema* in the River Lech is probably *Tychonema bornetii* due to its benthic occurrence. Trichome widths of *Tychonema bornetii* usually vary between 7.0 and 16 µm [25]. The DNA sequencing confirmed *Tychonema* to the genus level and indicated that it was only a single species and not several different *Tychonema* species. Nevertheless, morphological and molecular differentiation is difficult and sometimes ambiguous. The cells at the sampling sites in the southern part of the River Lech sometimes also showed morphological characteristics of *Phormidium*. Based on *16S rRNA gene* analyses, *Tychonema* and *Phormidium* are very similar and are assigned to a common lineage together with *Microcoleus* [26].

Literature on the occurrence of *Tychonema* is limited, though, in part, this may be due to the taxonomic separation of the genus *Tychonema* from *Oscillatoria* in 1988 by Anagnostidis and Komárek [27]. However, in recent years, the occurrence of *Tychonema* has been increasingly reported. A massive occurrence of tychoplanktic, ATX-producing *Tychonema* sp. has recently been described for Lake Tegel, Berlin, Germany [13]. As was the case with Mandichosee, the neurotoxicosis of several dogs attracted attention and led to further investigations. In Lake Tegel, *Tychonema* sp. was found primarily associated with water moss (*Fontinalis antipyretica*). Trichome widths of *Tychonema* sp. originating from Lake Tegel were narrower than those found in Mandichosee and ranged only between 6.8 and 8.9 µm. In contrast to Lake Tegel, the *Tychonema* found in Mandichosee and the River Lech was observed growing in the form of benthic mats also overgrowing various macrophytes (*Elodea*, *Myriophyllum* and *Potamogeton* species). 

*Tychonema* has also been found in the large lakes of the Southern Alps [28,29,30]. However, in contrast to the findings in Lake Tegel and Mandichosee, the pelagic form *Tychonema bourellyi* is present in these lakes [28]. 

*Tychonema* is known as cold-stenotherm genus with prevalence in clear water bodies in northern temperate regions [31]. The optimum growth temperature of a *T. bourellyi* strain from Cumbrian lakes was determined between 17 °C and 25 °C [32]. Comparably, *Tychonema* in Lake Tegel and Lake Garda show their growth maximum in spring at around 17 °C and disappear in summer with higher temperatures [13,29,30]. The water temperature in the River Lech and reservoir Mandichosee at *Tychonema*-positive sampling sites ranged between 11.8 and 19.8 °C. As the measurements started only after the dog casualties had occurred, the intensive growth phase may have already occurred when temperatures were higher. Monthly monitoring during bathing water surveillance revealed a maximum surface temperature of 24 °C in July 2019. It has already been presumed earlier that, in addition to temperature, the oligotrophication of lakes may play an important role, leading to shifts in cyanobacterial communities in favor of *Tychonema* species [15]. This is true for the lakes south of the Alps or Lake Tegel, but the water quality of mesotrophic Mandichosee did not change in recent years. It seems that *Tychonema* meets its requirement for cold, clear water bodies in middle Europe in spring in mesotrophic lakes and rivers or in rivers having their origin in mountains, but can also tolerate higher temperatures. 

High concentrations of ATXs (sum of dhATX and ATX) were found in the reservoir Mandichosee, with the proportion of dhATX usually accounting for more than 95%. Also in other producers such as *Oscillatoria*, *Cylindrospermum stagnale* and *Phormidium/Microcoleus*, the concentration of dhATX was shown to be much higher than that of ATX [26,33,34,35,36]. 

Toxins from benthic mats are usually related to biomass, however, risk assessment often requires information on toxin concentrations per liter of water. As benthic mats are not homogeneously distributed in the water body, such as planktic cyanobacteria, the determined toxin concentrations depend on the biomass of mats in a certain water volume. We have tried to simulate a possible up-take scenario and analyzed some parts of mat samples in a small volume of water (1 mL). Expressing the toxin concentration per liter then lead to extremely high concentrations of up to 68,000 ATXs µg/L, showing that large amounts of toxins can be taken up with a small volume of water when it contains a large mat fraction. ATX concentrations in the range of mg per liter have also been reported in tychoplanktic *Tychonema* and benthic *Phormidium* species [13,36]. 

ATX content in mat samples from Mandichosee were in the same mg per DW range as observed for *Cylindrospermum stagnale* (1200 µg dhATX/g DW) [35] and *Kamptonema* (*Phorm*.) *formosum* (8000 µg/g DW) [37]. 

The presence of anatoxins in mats and in the dogs’ stomachs indicates that they are the most likely reason for the neurotoxicosis of dogs at Mandichosee. The large amount of dhATX in the samples suggests this toxin to be the primary toxic agent, however, the data on the toxicity of dhATX in relation to ATX are contradictory. While earlier studies suggest that dhATX is about ten times less toxic than ATX [38,39], a more recent study shows that an intraperitoneal (i.p.) injection resulted in a lower toxicity of dhATX compared to ATX, while oral consumption caused greater toxicity [40]. Even if dhATX is less toxic than ATX, its high contents in *Tychonema* mats in Mandichosee can easily lead to the intake of lethal concentrations. High concentrations of ATXs in bathing waters such as in the reservoir Mandichosee are not only hazardous for dogs, but also for small children taking up mat material while playing with them or during bathing accidents. Adults are of lower risk as they would avoid swallowing mat material unless during near-drowning situations. In contrast to mammals, macrozoobenthos organisms such as *Chironomus* and *Deleatidium* larvae proved to be very resistant to high ATX concentrations (300–600 µg/L) and showed almost no mortality and no or only low accumulation of ATX [41,42]. Also the rainbow trout (*Oncorhynchus mykiss*) was not affected at similar concentrations but accumulated up to 23 µg ATX/g BW [43]. The consumption of such contaminated fish does not result in an acute dose but may exceed the tolerable daily intake concentration value (for lifetime consumption) of ATX [44]. 

The incidents at Mandichosee have shown that mass occurrences of benthic cyanobacteria cannot be detected with the current surveillance strategies of bathing waters. There are several reasons for this: When assessing the threat from cyanobacteria, the focus of monitoring is mainly on nutrient-rich stagnant and slow-flowing waters where primarily planktonic cyanobacteria may be present. The turbidity of the water body, determined as Secchi transparency, and cyanobacterial abundance (biovolume or cyano-chlorophyll-a) are the decisive parameters for more detailed analyses and, if indicated, for the decision to prohibit bathing. Special attention is given to surface accumulations of cyanobacteria. Thus, benthic mass occurrences of cyanobacteria are not surveyed and are usually only discovered after animal fatalities. To our knowledge, only New Zealand currently includes surveying benthic cyanobacteria in their guidelines for recreational fresh waters [45]. 

However, observations in recent years indicate an increase in potential toxic benthic cyanobacteria, even in German lakes. The reason for this phenomenon is not clear. Possible reasons are higher transparency due to improved water quality, as has also been described for Lake Tegel in recent years [46] or due to sinking water levels in dry summers. The fact that the incidents have occurred mainly in recent years suggest that they might be directly or indirectly connected to climate change. The results of the present study indicate that not only Mandichosee is affected by the toxic benthic *Tychonema* occurrence, but also other reservoirs upstream and downstream, since it is a system connected by the River Lech.

## 4. Conclusions

Based on the preliminary findings, a systematic monitoring of the *Tychonema* occurrence at the Lech reservoirs is proposed. If dog deaths had not occurred at Lake Tegel and the reservoir Mandichosee, the occurrence of the toxin producer *Tychonema* would almost certainly not have been detected. It cannot be excluded that *Tychonema* is already present in more lakes than is currently known and has not yet been detected due to lack of knowledge and insufficient monitoring strategies. Literature on the conditions for *Tychonema* occurrence is very limited to date. Therefore, it is important to investigate the distribution and growth conditions of the toxin-producing cyanobacterium *Tychonema* in detail, to focus on the investigation of toxic benthic cyanobacteria and to develop appropriate surveillance strategies.

## 5. Materials and Methods 

### 5.1. Study Site

The investigations have focused on the reservoir Mandichosee. This reservoir is situated in Southern Bavaria, Germany, near the city of Augsburg (48°15′45″ N. 10°56′0″ E). With a surface area of 1.6 km^2^, it is the second largest of the numerous reservoirs of the River Lech. Due to its proximity to a metropolitan area, Mandichosee is a very popular lake, which is used intensively for leisure activities and recreation. According to the local district authorities, Mandichosee has been known for excellent bathing water quality. 

With a length of 256 km, the River Lech is one of the largest and most important rivers in Bavaria. The River Lech has its source in Vorarlberg, Austria, and streams through South Bavaria before it flows into the River Danube (Bavaria, Germany). As a typical alpine river, the River Lech was originally characterized by early summer floods due to snow melting and since precipitation in the Alpine region is very high in summer, flood events could theoretically occur at any time. As in all alpine rivers, the water temperature was mostly low, oxygen content and flow velocity high. To protect against flooding by regulating the water level and to generate electricity by hydro power, 23 dams and a cascade of reservoirs were built along the River Lech over a distance of about 90 km from 1954 to 1983. In the south, the largest and highest situated reservoir, the Forggensee, was first put into operation, and successively the other ones, of which the Mandichosee is the most northerly. These reservoirs have a great influence on the temperature, the flow velocity and the bed load of the River Lech. Because of this, it is considered to be the most modified river in Bavaria today. Along the River Lech, there are several protected areas for the use of drinking water supply. Various reservoirs serve for leisure and recreational purposes.

### 5.2. Animal Necroscopy and Histopathology

Two of the accidentally intoxicated dogs found along the reservoir Mandichosee (D1: Husky, adult, spayed; D2: Yorkshire terrier, juvenile, male) were submitted to the Bavarian Health and Food Safety Authority for necropsy. Formalin-fixed specimens (brain, bone marrow, heart, lung, kidney, liver, intestine) were processed routinely for histological investigation (paraffin embedded, cut in 4 µm thick sections, mounted on glass slides and stained with hematoxylin and eosin). In addition, formalin-fixed frozen tissue (liver) was stained with Sudan red (14 µm thick sections, mounted on glass slides). 

In the third case (D3: Jack Russell terrier, juvenile, male), necropsy was conducted at the Institute of Veterinary Pathology of the University of Munich according to a standardized post-mortem examination protocol. For histopathological examination, tissue samples of several organs were taken considering a defined collection protocol. With reference to the preliminary report (acute seizures, oral discharge), a special neuropathological examination was performed according to directives of the International Veterinary Epilepsy Task Force (IVETF). The formalin-fixed (7% neutral-buffered formaldehyde) and paraffin-embedded samples were routinely processed and stained with hematoxylin and eosin as well as Giemsa stain. 

After the histopathological examination, the stomach contents of D1 and D3 were sent from the Bavarian Health and Food Safety Authority (LGL) to the German Environment Agency (UBA) for toxin analyses by LC-MS/MS.

### 5.3. Sampling and in Situ Measurements of Hydro-physical Parameters

Sampling and in situ measurements at the reservoir Mandichosee were carried out on 19 August, 21 August and 10 September, at the reservoirs Kaufering (18), Schwabstadl (19), Scheuring (20), Prittriching (21) and Unterbergen (22) on 12 September, and at the reservoirs Forggensee (0), Roßhaupten (1), Prem (2), Lechaue (2a), Lechbruck-Urspring (3), Kreut (5), Dornau (6), Apfeldorf (9) and Pitzling (14) on 19 September 2019. 

Samples were taken several times at different locations along the shoreline of the reservoir Mandichosee, specifically in the bathing area (23.1) where the dog casualties occurred (see introduction), and once at each location of the other reservoirs (Figure 2).

At each sampling, benthos from different substrates (sediment, stones, wood), detached benthos floating as tychoplanktic material and macrophytes, e.g., *Elodea*, *Myriophyllum*, were collected for microscopic and molecular analysis. For further molecular analyses, 0.5 L of water were taken from the benthic or floating material, from the plant stock and from the adjacent free water. In addition, 0.5 L water were taken at selected sampling sites for hydrochemical analyses. During each sampling campaign, the hydro-physical parameters, i.e., temperature, pH value, conductivity and oxygen saturation, were measured directly on site with a multi-parameter probe (MPP 930 IDS, WTW, Weilheim, Germany). 

Additional samples for toxin measurements were taken on 14, 21, 23 August and 17 September from Mandichosee and on 17 September from reservoirs 18–22. The samples originate from different sample types (water, algal mat, macrophytes). The exact sample origins can be found in Table 1. 

### 5.4. Post-Processing of the Samples

The water samples for the molecular analysis were filtered through 0.2 µm filters (Sartorius, Göttingen, Germany), and stored at −20 °C until DNA extraction. 

The red-brown biofilms of benthic mats and macrophytes were isolated with a sterile inoculation loop, transferred into 1.5 mL Eppendorf tubes and were stored at −20 °C until DNA extraction. 

For toxin analysis, sub-samples were mostly taken from well-mixed water, water/mat or water/macrophyte samples. In order to relate ATX contents to fresh weight, a few sub-samples were prepared by weighing around 100 mg fresh mat material and adding 1 mL of 0.1% formic acid. All sub-samples were frozen prior to analysis. 

### 5.5. Toxin Analyses

Analysis of CYN, deoxy-CYN, ATX and HATX as well as dhATX and dhHATX, was carried out as detailed previously [13]. Briefly, water samples with or without parts of *Tychonema* mats or macrophytes covered with *Tychonema* and the stomach contents of the dogs were acidified with formic acid to a final concentration of 0.1% formic acid and frozen/thawed twice. The samples were ultrasonicated for 10 min, shaken for 1 h, centrifuged and filtered (0.2 µm, PVDF, Whatman, Maidstone, UK) before analysis by LC-MS/MS.

LC-MS analysis was carried out on an Agilent 2900 series HPLC system (Agilent Technologies, Waldbronn, Germany) coupled to an API 5500 QTrap mass spectrometer (AB Sciex, Framingham, MA, USA) equipped with a turbo-ionspray interface. Chromatographic separation of 10 µl crude extracts was performed on an Atlantis C18 column (2.1 mm, 150 mm, Waters, Eschborn, Germany) at 30 °C. Compounds were eluted at a flow rate of 0.25 mL min^−1^ using a linear gradient of 0.1% formic acid in water (A) and 0.1% formic acid in methanol (B) from 1% to 25% B within 5 min. 

Identification of CYN, deoxy-CYN, ATX and HATX was performed in the positive multiple reaction monitoring (MRM) mode with the following transitions: CYN *m/z* 416.1 [M + H]+ to 194 and 416.1/176, deoxy-CYN *m/z* 400.1 [M + H]+ to 320 and 400.1/194, ATX *m/z* 166.1 [M + H]+ to 149, 166.1/131, 166.1/91, 166.1/43 and HATX *m/z* 180.0 [M + H]+ to 163, 180.0/145 and 180.0/91. CYN and ATX were from the National Research Council (Canada) and deoxy-CYN and HATX were from Novakits (Nantes, France). The detection limit for CYN and deoxy-CYN was 0.02 µg L^−1^, for ATX 0.02 µg L^−1^ and for HATX 0.04 µg L^−1^.

In addition, dihydroanatoxin-a (DhATX) and dihydrohomoanatoxin-a (DhHATX) were analyzed using fragment ions described in the literature as no reference material was available [47]. DhATX was quantified with ATX using the fragment *m/z* 43 present in both substances as a quantifier ion. 

### 5.6. Microscopy

Algae or cyanobacteria trichomes were isolated immediately after sampling in the laboratory with a sterile inoculation loop from the benthic mats, from the floating material or from the macrophytes and transferred to microscopic slides. The subsequent analysis was performed on a microscope (Leica DMRBE, Leica, Wetzlar, Germany) with photographic equipment (Zelos 285 GV, Kappa, Norderstedt, Germany). Potential *Tychonema* trichomes were measured, morphologically characterized and photographed.

### 5.7. Molecular Approach

#### 5.7.1. DNA Extraction and Purification

DNA was extracted from the filters or the Eppendorf tubes using the phenol/chloroform-based method described by Zwirglmaier et al. [48]. Subsequently, DNA was purified using Sure Clean Plus (Bioline, London, UK) and eluted in sterile ultrapure water. The DNA was then stored frozen until further analysis.

#### 5.7.2. Polymerase Chain Reaction

Polymerase chain reaction was performed to detect the target genes for ATX, microcystin (MCY) and saxitoxin (SXT). Primer pairs for the target genes *anaC*, *mcyE* and *sxtA* were anxgen-F and anxgen-R [21], mcyE-F2 and mcyE-R4 [49] and sxtA-F and sxtA R [50]. The strain PCC6506 (*Oscillatoria* sp., obtained from the Pasteur Culture Collection of Cyanobacteria, Paris, France) served as positive control for *anxC*, strain SAG14.85 (*Microcystis aeruginosa*, obtained from the Culture Collection of Algae at Göttingen University, Göttingen, Germany) served as positive control for *mcyE* and strain NIVA-CYA655 (*Aphanizomenon gracile*, obtained from the Norwegian Culture Collection of Algae, Oslo, Norway) as positive control for *sxtA*. The cycling conditions in the CFX thermocycler (CFX Connect, Biorad, Germany) were as follows: 94 °C for 10 min, 30 cycles of 94 °C for 30 s, annealing temperature for 30 s and 72 °C for 30 s, followed by a final elongation step of 72 °C for 7 min. Annealing temperatures were 55.8 °C for *anaC*, 56 °C for *mcyE* and 57.5 °C for *sxtA*.

#### 5.7.3. Sequencing

Samples were bidirectionally sequenced at the Core Facility Microbiome, ZIEL, TUM, Freising using Illumina MiSeq v3 2 × 300 paired-end sequencing. Polymerase chain reaction (PCR) primers used for the first step were S-DBact-0341-b-S-17 (5′ *TCGTCGGCAGCGTCAGATGTGTATAAGAGACAG*CCTACGGGNGGCWGCAG 3′) and S-D-Bact-0785-a-A-21 (5′ *GTCTCGTGGGCTCGGAGATGTGTATAAGAGACAG*GACTACHVGGGTATCTAATCC 3′) (Illumina overhang adapter in italics). These primers cover the *16S rRNA gene* variable regions V3-V4. These hypervariable regions combined with a paired-end sequence configuration are recommended as the most effective study design [51]. Processing of Illumina MiSeq sequence data was done within IMNGS [52]. The operational taxonomic units (OTUs) were clustered at 97% sequence identity and subsequently classified with SINA online [53]. For classification, SILVA taxonomy [23] was used, which was implemented in SINA online. Sequencing was carried out in two runs, which were analyzed separately. Sequence data have been submitted to NCBI’s Sequence Reads Archive (BioProject IDs PRJNA671879 and PRJNA672047). 

### 5.8. Hydrochemical Analysis

The water samples for hydrochemical analysis were filtered through a 0.2 µm pore size membrane filter (GE Healthcare, Amersham, UK) prior to measurement of nitrate–nitrogen (NO_3_^−^-N) and ammonium–nitrogen (NH_4_^+^-N) concentrations. Total phosphorus (TP) concentration was calculated from unfiltered water samples. Values of TP and NH_4_^+^-N were determined following established methods by the German Chemists’ Association [54]. NO_3_^−^-N values were determined using the method described by [55]. 

## Figures and Tables

**Table 1:** ATX and DhATX concentrations in samples from Mandichosee, other Lech reservoirs and the investigated dogs.

Sample Origin (Site)	Sample	Date (Day.Month.Year)	ATX (µg/L)	DhATX (µg/L)
Dog (D3)	Stomach content	14 August 19	n.d.	563
Dog (D1)	Stomach content	14 August 19	25.2	1182
Reservoir Mandicho (23.1)	Mat from shoreline	21 August 19	24.4–320	1917–39,528
Reservoir Mandicho (23.1)	Floating mat	21 August 19	241–453	16,954–67,622
Reservoir Mandicho (23.2)	Mat from shoreline	21 August 19	28.2–214	2375–23,488
Reservoir Mandicho (23.2)	Water and macrophytes	14 August 19	9.9–40.9	607–3012
Reservoir Mandicho (23.2)	*Elodea* with water	21 August 19	10.3–12.9	320–326
Reservoir Mandicho (23.2)	Macrophytes with water	21 August 19	n.d.	0.7
Reservoir Mandicho (23.2)	*Myriophyllum* with water	21 August 19	3.5	157
Reservoir Mandicho (23.1)	Macrophytes/Periphyton	23 August 19	7.7–272	3113–29,335
Reservoir Mandicho (23.1)	Macrophytes and water	17 September 19	2.1	310
Reservoir Mandicho (23.1)	Macrophytes and water	17 September 19	0.1	0.2
Reservoir Mandicho (23.2)	Water surface above mat	21 August 19	<LOQ	2.7
Reservoir Mandicho (23.2)	Water surface 20 cm above mat	21 August 19	<LOQ	1.0
Reservoir Mandicho (23.2)	Water surface 50 cm above mat	21 August 19	<LOQ	1.4
Reservoir Mandicho (23.2)	Water surface 100 cm above mat	21 August 19	<LOQ	5.0
Reservoir Mandicho (23.2)	Water	14 August 19	<LOQ	3.8
Reservoir Mandicho (23.1)	Water	21 August 19	n.d.	0.6
Reservoir Prittriching (21)	Water	17 September 19	n.d.	n.d.
Reservoir Scheuring (20)	Water	17 September 19	n.d.	n.d.
Reservoir Schwabstadl (19)	Water	17 September 19	n.d.	0.7
Reservoir Kaufering (18)	Water	17 September 19	n.d.	n.d.

LOQ: Limit of Quantification.

## Supplementary Materials

The following are available online at https://www.mdpi.com/2072-6651/12/11/726/s1, Table S1: Sample overview and results of microscopy, hydro-physical and hydro-chemical measurements as well as molecular analyses, Table S2: ATX and DhATX contents in mat material from Mandichosee.
