# Supplementary material for: Mass Occurrence of Anatoxin-a- and Dihydroanatoxin-a-Producing Tychonema sp. in Mesotrophic Reservoir Mandichosee (River Lech, Germany) as a Cause of Neurotoxicosis in Dogs
Source: Toxins (Basel). 2020 Nov 20;12(11):726. doi: 10.3390/toxins12110726 (PMC7699839; doi:10.3390/toxins12110726)
Supplement: Supplementary file 1 [file toxins-12-00726-s001.pdf]

Supplementary Materials: Mass Occurrence of Anatoxin-a- and Dihydroanatoxin-a-Producing *Tychonema* sp. in Mesotrophic Reservoir Mandichosee (River Lech, Germany) as a Cause of Neurotoxicosis in Dogs

Franziska Bauer, Jutta Fastner, Bernadett Bartha-Dima, Wolfram Breuer, Almuth Falkenau, Christian Mayer and Uta Raeder

Table S1. Sample overview and results of microscopy, hydro-physical and hydro-chemical measurements as well as molecular analyses.

Sample origin (site)	Date (Day.Month.Year)	Microscopy (T = *Tychonema*)	Temperature [°C]	pH	Conductivity [µS/cm]	Oxygen saturation [%]	Oxygen content [mg/L]	Total phos-phorus [µg/L]	Sequencing (T = *Tychonema*)	*anaC* (yes/no)
Mandichosee (23.2)	19.08.19	T	n.d.	n.d.	n.d.	n.d.	n.d.	n.d.	T	Y
Mandichosee (23.3)	19.08.19	-	19.8	8.7	311	142	12.2	n.d.	T	Y
Mandichosee (23.1)	21.08.19	T	19.3	8.7	314	115	10.1	n.d.	T	Y
Mandichosee (23.2)	21.08.19	T	19.6	8.7	308	119	10.3	11.1	T	Y
River Lech downstream	21.08.19	-	n.d.	n.d.	n.d.	n.d.	n.d.	11.5	T	N
Mandichosee (23.1)	10.09.19	T	n.d.	n.d.	n.d.	n.d.	n.d.	n.d.	T	N
Mandichosee (23.2)	10.09.19	T	14.3	8.4	341	n.d.	10.3	n.d.	T	Y
Mandichosee (23.3)	10.09.19	T	14.3	8.6	307	118	10.4	n.d.	-	N
Unterbergen (22)	12.09.19	-	16.1	8.8	341	131	12.5	20.3	T	N
Prittriching (21)	12.09.19	T	15.8	8.8	337	129	12.1	13.4	T	Y

Scheuring (20)	12.09.19	T	17.7	9.4	324	177	17.0	15	T	N
Schwabstadl (19)	12.09.19	T	19.3	9	327	142	12.3	16.1	T	Y
Kaufering (18)	12.09.19	T	17.3	9	324	146	13.5	17.3	T	N
Landzunge Pitzling (14)	19.09.19	-	16.7	8.7	351	133	12.1	n.d.	-	N
Apfeldorf (9)	19.09.19	-	15.6	8.7	351	129	11.9	n.d.	T	N
Kreut (5)	19.09.19	-	15.2	9.0	332	144	13.4	n.d.	T	N
Lechbruck-Urspring (3)	19.09.19	T	15.6	8.9	315	145	13.2	n.d.	T	N
Lechaue (2A)	19.09.19	T	11.8	8.1	494	96	9.5	n.d.	T	N
Prem (2)	19.09.19	-	13.3	8.5	332	104	10.0	n.d.	T	N
Roßhaupten (1)	19.09.19	-	14.6	n.d.	322	105	9.8	n.d.	-	N
Forggensee (0)	19.09.19	-	15.9	8.8	318	107	9.7	n.d.	-	n.d.

Table S2. ATX and DhATX contents in mat material from reservoir Mandichosee.

**Sample origin (site)**	**Sample**	**Date (Day.Month.Year)**	**ATX [µg/g FW]**	**dhATX [µg/g FW]**
Mandichosee (Z)	Mat from shoreline	21.8.19	2.7	329
Mandichosee (Z)	Mat from shoreline	21.8.19	2.5	288
Mandichosee (Z)	Floating mat	21.8.19	2.8	231
Mandichosee (Z)	Floating mat	21.8.19	0.5	99
Mandichosee (Y)	Mat from shoreline	21.8.19	3.2	350
Mandichosee (Y)	Mat from shoreline	21.8.19	0.8	85.2

FW: fresh weight.
